# Resistance Profiles and Virulence Determinants in Biofilm-Forming *Enterococcus faecium* Isolated from Raw Seafood in Bangladesh

**DOI:** 10.3390/pathogens12091101

**Published:** 2023-08-28

**Authors:** Md. Ashek Ullah, Md. Saiful Islam, Md. Liton Rana, Farhana Binte Ferdous, Fahim Haque Neloy, Zannatul Firdous, Jayedul Hassan, Md. Tanvir Rahman

**Affiliations:** Department of Microbiology and Hygiene, Faculty of Veterinary Science, Bangladesh Agricultural University, Mymensingh 2202, Bangladesh; ashek.21110216@bau.edu.bd (M.A.U.); dvm41257@bau.edu.bd (M.S.I.); liton.21110215@bau.edu.bd (M.L.R.); farhanaferdous1501184@gmail.com (F.B.F.); neloymb002@gmail.com (F.H.N.); alaila796@gmail.com (Z.F.); dr_jahid@bau.edu.bd (J.H.)

**Keywords:** seafood, *Enterococcus faecium*, biofilm formation, antibiotic resistance, virulence determinants, multidrug resistance, multiple antibiotic resistance, Bangladesh

## Abstract

Pathogenic, antibiotic-resistant, and biofilm-forming bacteria can be transferred to humans through the consumption of contaminated seafood. The present study was carried out to determine antibiotic resistance profiles and virulence determinants in biofilm-forming *Enterococcus faecium* isolated from seafood in Bangladesh. A total of 150 seafood samples, including shrimp (n = 50), crabs (n = 25), and marine fish (n = 75), were screened using cultural, staining, biochemical, polymerase chain reaction (PCR), Congo red (CR), and disk diffusion (DD) assays. In PCR, *E. faecium* was detected in 27.3% (41/150; CI_95%_ 20.8; 34.9) of samples, where marine fish (34.7%, CI_95%_ 24.9; 45.9) had the highest prevalence (*p* < 0.05) compared to crabs (32%, CI_95%_ 17.2; 51.6) and shrimp (14%, CI_95%_ 7.0; 26.1). Thirty-two (78.1%, CI_95%_ 63.3; 88.0) of the *E. faecium* isolates were determined to be biofilm formers in the CR test, where 43.9% (18/41, CI_95%_ 29.9; 59.0) and 34.2% (14/41, CI_95%_ 21.6; 49.5) of the isolates were strong and intermediate biofilm formers, respectively. In PCR, virulence genes, i.e., *pil* (100%), *ace* (92.7%), *agg* (68.3%), *fsrA* (65.9%), *gelE* (63.4%), *sprE* (53.7%), *fsrB* (51.2%), and *fsrC* (43.9%), were detected in *E. faecium* isolates. All the *E. faecium* isolates were phenotypically resistant to ≥3 antimicrobial categories and ≥3 antibiotics, including WHO-classified reserve antibiotics linezolid (70.7%) and fosfomycin (19.5%). Moreover, the multiple antibiotic resistance index ranged up to 0.8, showing resistance to ten antibiotics and eight antibiotic classes. In this study, the prevalence of virulence genes and antibiotic resistance was significantly greater (*p* < 0.05) in strong biofilm-forming *E. faecium* strains as compared to strains with intermediate and non-biofilm-forming abilities. As far as we know, this study, for the first time in Bangladesh, determined antibiotic resistance and detected virulence genes in biofilm-forming *E. faecium* isolated from seafood samples. The data from this study could play a significant role in evaluating potential health hazards linked to the ingestion of uncooked or minimally processed seafood.

## 1. Introduction

Bangladesh possesses significant potential for both catch fisheries and aquaculture because of its extensive network of inland lakes and rivers. With the world’s largest flooded wetland, and ranking as Asia’s third-largest hub of aquatic biodiversity after China and India, Bangladesh is highly regarded as a prime global location for fishing [1]. Consumption of seafood has been on the rise in Bangladesh. Furthermore, the development of marine fish production in the country has made a substantial contribution to its overall fisheries output, constituting around 15% of the total 4.8 million metric tons [2]. However, the significant nutritional content of sea products makes them an important carrier of pathogenic bacteria [3].

Seafood possesses the ability to transport enterococci from various origins such as rivers, lakes, and ocean water, which are regularly exposed to urban wastewater and water from aquaculture that may be contaminated. The presence of enterococci in foods, including raw food, is primarily due to their ability to thrive in challenging environmental conditions associated with food production and storage [4]. Furthermore, these bacteria are widely distributed and can be commonly found in both saltwater and seafood [5]. Enterococci, including *Enterococcus faecium* and *E. faecalis*, are increasingly emerging as important pathogens for humans [6]. They are recognized as a major contributor to hospital-acquired infections. About 90% of clinical infections and over 10% of hospital-acquired infections in humans are attributed to *E. faecium* and *E. faecalis* [7]. Interestingly, *E. faecium* ranks as the fourth most prevalent human pathogen worldwide [8].

Bacteria frequently utilize biofilms as a tactic to endure difficult environmental situations. These biofilms develop when groups of microbial cells cluster together and are surrounded by extracellular polymeric substances. In contrast to solitary planktonic cells, bacteria that create biofilms possess numerous benefits, including enhanced resistance to hostile environmental circumstances, disinfectants, and antimicrobial agents [9,10]. Enterococci are recognized for their capacity to develop biofilms, a phenomenon wherein groups of cells tightly attach to a variety of surfaces, encompassing both animate and inanimate substrates [11]. Moreover, studies have indicated that certain genes associated with virulence, such as *fsrA*, *fsrB*, and *fsrC* (quorum-sensing genes), *pil* (pili gene), *gelE* (gelatinase gene), *sprE* (serine protease gene), *ace* (gene connected to enterococcal adhesion to collagen), *agg* (aggregation substance gene), and *cyl* (cytolysin gene), play an important role in the pathogenicity of enterococci by allowing the microbes to bind to target cells and facilitating genetic material transfer between cells [12].

The escalating issue of antimicrobial resistance (AMR) is a notable and increasing worry in the realm of public health, posing a significant barrier to both human and veterinary medicine [13]. The rising occurrence of AMR is progressively causing heightened apprehension within public health circles due to its ability to undermine the effectiveness of antibiotics and complicate the management of bacterial infections [14]. Enterococci inherently display resistance to multiple antimicrobial agents typically employed for addressing infections caused by Gram-positive microorganisms. Furthermore, the capacity of enterococci to gain antibiotic resistance by means of plasmid and transposon transfer, exchange of chromosomal material, or mutation is also noteworthy [15]. The notable rise of bacteria resistant to multiple antibiotics detected in diverse aquatic environments could hold substantial implications for public health, given that treating human infections caused by these organisms might become challenging using pharmaceutical interventions. In recent years, there has been a growing focus on *E. faecium*, mainly because of its ability to acquire multiple determinants for antibiotic resistance, a trait that sets it apart from other fecal bacteria released into the environment [16].

In contrast to various food origins, limited data are accessible concerning AMR and virulence in biofilm-forming *E. faecium* detected from seafood in Bangladesh. The present study was carried out to address the aforementioned gaps since, as far as we know, there are no data in Bangladesh concerning the antibiotic resistance and virulence characteristics of biofilm-forming *E. faecium* in seafood.

## 2. Materials and Methods

### 2.1. Sampling Sites

**Figure 1:**
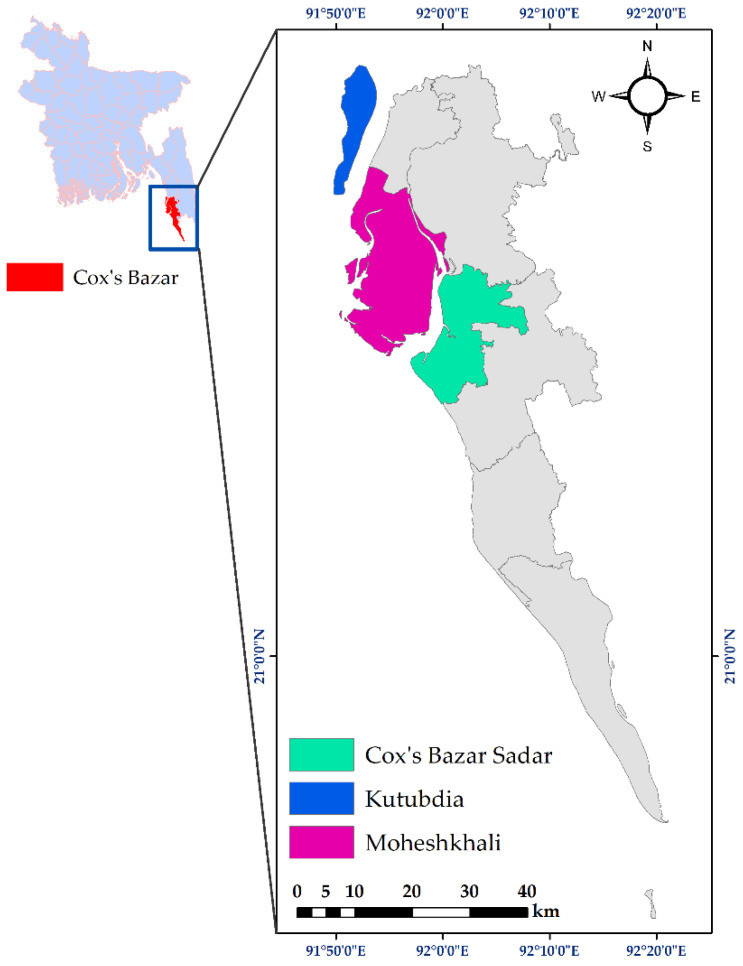
The map of the sampling sites during this study. The study area map was prepared using ArcMap.v.10.7 (ArcGIS Enterprise, ESRI, Redlands, CA, USA).

The samples were collected during the period of January 2022 to March 2023 from three coastal areas, i.e., Cox’s Bazar Sadar (21.4272° N, 92.0061° E), Moheshkhali (21.5374° N, 91.9418° E), and Kutubdia (21.8167° N, 91.8583° E) Upazilas (Figure 1) in the Chattogram region of Bangladesh. These areas were selected based on different factors, such as the availability of seafood in those areas, easy access to seafood, and their high demand in those areas.

### 2.2. Sample Collection and Processing

In this study, a total of 150 raw seafood samples, including shrimp (n = 50), crab (*Scylla serrata*, n = 25), and sea fish (n = 75), were collected. Shrimp included white shrimp (*Penaeus indicus*, n = 25) and tiger shrimp (*Penaeus monodon*, n = 25), while the fish species constituted rupchanda (*Pampus chinensis*, n = 25), tuna (*Euthynnus affinis*, n = 25), and loitta (*Harpadon nehereus*, n = 25). During the study duration, sampling visits were carried out every two weeks in various retailed fish markets, and each sample was promptly placed into a sterile zipper bag, followed by keeping them on ice for transportation to the Microbiology laboratory, Department of Microbiology and Hygiene, BAU, Mymensingh (24.7245° N, 90.4372° E). In the laboratory, the samples’ outer surface was treated with 70% (*v*/*v*) alcohol for cleaning purposes. For sea fish, each sample weighing 25 g was homogenized with 225 mL of buffered peptone broth (BPB) (HiMedia, Mumbai, Maharashtra, India), and the mixture was left to incubate overnight at 37 °C [15]. For shrimp and crabs, the brain, leg, muscle, and intestine from each sample were blended, and 25 g of the resulting blend was homogenized and incubated using similar procedures [17].

### 2.3. Isolation and Molecular Detection of Enterococcus faecium

The initial isolation of *E. faecium* was performed by culturing the bacterial sample on an enterococcus agar base (EAB) (HiMedia, Mumbai, Maharashtra, India) medium. A single loopful of the bacterial cultured broth was streaked on EAB medium and then incubated aerobically overnight at 37 °C. Colonies appearing as white or pale pink on the EAB medium were initially considered to be *E. faecium*. Subsequently, the presumed colonies underwent Gram staining and various biochemical tests, including sugar fermentation, Voges–Proskauer, indole, and catalase tests [18]. Finally, *E. faecium* isolates were analyzed using a simplex polymerase chain reaction (PCR) to detect the presence of *E. faecium* by targeting the *ddl_E_. _faecium_* gene (Table 1).

The PCR procedure involved obtaining genomic DNA from isolated *E. faecium* using the boiling method, following the methodology previously described [21]. In brief, 1 mL of the enriched culture underwent centrifugation at 5000 rpm for 5 min. After discarding the supernatant, a suspension was formed by adding 200 μL of phosphate buffer solution (PBS). The subsequent steps encompassed boiling the suspension and allowing it to cool for 10 min at each step. This was followed by another round of centrifugation at 10,000 rpm for 10 min. The resultant supernatant, which held the genomic DNA, was collected and preserved at a temperature of −20 °C to facilitate future analysis.

Each PCR reaction was conducted within a final volume of 20 µL (nuclease-free water: 3 µL; 2X master mixture (Promega, Madison, WI, USA): 10 µL; forward and reverse primers: 1 µL each; and DNA template: 5 µL). The PCR-positive controls encompassed *E. faecium* genomic DNA that had previously tested positive for the relevant gene. For PCR-negative controls, non-template controls were implemented, employing PBS instead of genomic DNA.

Once the amplification process was completed, the PCR outputs underwent electrophoresis on a 1.5% agarose gel. This gel was treated with ethidium bromide for staining purposes and then visualized using an ultraviolet transilluminator (Biometra, Göttingen, Germany). As a point of reference to confirm the anticipated sizes of the amplified PCR products, a 100 bp DNA ladder (Promega, Madison, WI, USA) was employed.

### 2.4. Determination of Biofilm-Forming Abilities of Enterococcus faecium

Biofilm formation in *E. faecium* was characterized phenotypically using the Congo Red (CR) test, following the methodology previously outlined [22]. The CR test involved culturing the *E. faecium* isolates on Congo Red Agar (CRA) plates. Initially, *E. faecium* cultures were streaked on CRA plates and then incubated at 37 °C for 24 h. Based on the colony appearance on the CRA plates, isolates exhibiting dry filamentous crusty black colonies, pink colonies with a dark center, and smooth pink colonies were classified as strong, intermediate, and non-biofilm producers, respectively [10].

### 2.5. Molecular Detection of Virulence Genes in Enterococcus faecium

The presence of virulence-related genes commonly observed in *E. faecium*, such as *fsrA*, *fsrB*, *fsrC*, *pil*, *gelE*, *sprE*, *ace*, *agg*, and *cyl*, was identified using a simplex PCR assay (Table 1). The PCR protocols used to detect these genes were the same as those described earlier in Section 2.3. Moreover, the PCR-positive controls encompassed *E. faecium* genomic DNA that had previously tested positive for the relevant virulence genes. For PCR-negative controls, non-template controls were implemented, employing PBS instead of genomic DNA.

### 2.6. Antibiotic Susceptibility Test

As per the Clinical and Laboratory Standards Institute (CLSI) guidelines [23], the disk diffusion method [24] was performed to determine the antibiotic sensitivity patterns of *E. faecium* isolates. To conduct the antibiotic susceptibility test (AST), bacterial colonies were grown on EAB agar plates at 37 °C for 18–24 h. A suspension of 2–3 bacterial colonies was then prepared in 0.85% sterile normal saline solution and adjusted to a final concentration of 0.5 McFarland standard units. After an additional 24 h incubation at 37 °C, the bacterial inoculum was spread onto Mueller–Hinton agar plates using sterile cotton swabs, and finally, previously selected antibiotics were dispensed onto the surface of the inoculated agar plate. In this study, we selected thirteen commercially available antibiotics (HiMedia, Mumbai, Maharashtra, India) belonging to ten different antibiotic classes. The three World Health Organization (WHO)-classified antibiotic groups, i.e., access, watch, and reserve, were also focused on during the antibiotic selection. Access antibiotics included penicillins (penicillin—10 μg and ampicillin—10 μg), tetracyclines (tetracycline—30 μg), nitrofurans (nitrofurantoin—300 μg), and amphenicols (chloramphenicol—30 μg); watch antibiotics were glycopeptides (vancomycin—30 μg), macrolides (erythromycin—15 μg), fluoroquinolones (ciprofloxacin—5 μg, levofloxacin—5 μg, and norfloxacin—10 μg), and ansamycins (rifampin—5 μg); and reserve antibiotics included phosphonic acids (fosfomycin—200 μg) and oxazolidinones (linezolid—30 μg). The bacterial strain *Escherichia coli* ATCC25922 was employed as the designated control strain. Isolates that displayed resistance to at least three antimicrobial categories were classified as multidrug-resistant (MDR) [25]. The following formula [26] was utilized to calculate the multiple antibiotic resistance (MAR) indices:

$$\mathrm{MAR}\;\mathrm{index}=\frac{The\;count\;of\;antibiotics\;to\;which\;an\;isolates\;showed\;resistance}{The\;total\;number\;of\;antibiotics\;employed\;in\;this\;study}$$

### 2.7. Statistical Analysis

The data acquired from this study were inputted into Excel 365 (Microsoft/Office 365, Redmond, WA, USA) and exported to the Statistical Package for Social Science (SPSS.v.25, IBM, Chicago, IL, USA) and GraphPad Prism (Prism.v.8.4.2, San Diego, CA, USA) for further analyses.

A descriptive analysis was undertaken to ascertain the prevalence of different variables associated with the *E. faecium* isolates. To estimate the prevalence, a binomial 95% confidence interval (CI) was calculated using a previously established method [27] implemented in GraphPad Prism. The chi-square test for relatedness (Z-test for proportion) was used to identify any sample-wise variations in the frequencies of *E. faecium* isolates and among different types of biofilm-forming *E. faecium* isolates. Additionally, a similar test was performed to determine any relatedness between the various degrees of biofilm formation among *E. faecium* isolates and the occurrence of virulence genes and phenotypic antibiotic resistance. Statistical significance was denoted by a *p*-value below 0.05 (*p* < 0.05). Furthermore, a bivariate analysis using SPSS was conducted to explore the potential association between any of the two virulence genes of *E. faecium* isolates, as well as their resistance to any two antibiotics. A *p*-value less than 0.05 was considered statistically significant in this bivariate analysis.

## 3. Results

### 3.1. Prevalence of Enterococcus faecium

**Figure 2:**
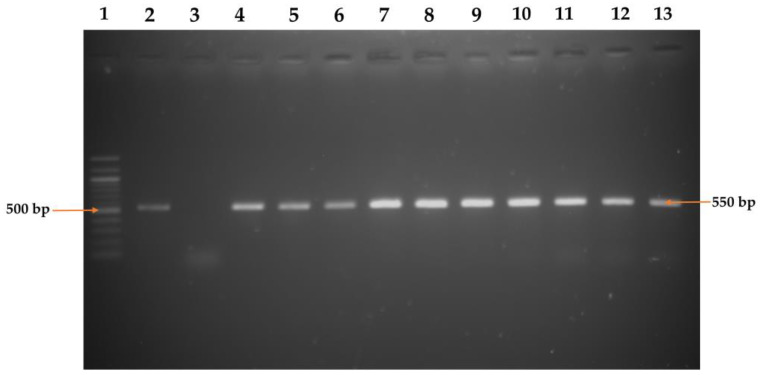
PCR amplification of *ddl_E_. _faecium_* gene of *Enterococcus faecium*. Lane 1: 100 bp DNA Marker; Lane 2: Positive control; Lane 3: Negative control; and Lane 4–13: Representative *ddl_E_. _faecium_* gene of *Enterococcus faecium* isolates.

Out of 150 samples, 53 (35.3%, 95% CI: 28.1–43.3%) were found to be positive for *E. faecium* based on colony characteristics of selective media, staining character, and biochemical properties. In PCR targeting of the *ddl_E. faecium_* gene (Figure 2), 27.3% (41/150, 95% CI: 20.8–34.9%) of raw seafood samples contained *E. faecium*, where a significantly (*p* < 0.05) higher prevalence of the isolates was exhibited in marine fish (34.7%, 95% CI: 24.9–45.9%) compared to crab (32%, 95% CI: 17.2–51.6%) and shrimp (14%, 95% CI: 7.0–26.1%) samples (Table 2).

### 3.2. Biofilm-Forming Ability of Isolated Enterococcus faecium

In the Congo Red test, 43.9% (18/41, 95% CI: 29.9–59.0%) of the *E. faecium* isolates exhibited strong biofilm formation, while 34.2% (14/41, 95% CI: 21.6–49.5%) of the isolates displayed intermediate biofilm formation, and the remaining 21.9% (9/41, 95% CI: 12.0–36.7%) were classified as non-biofilm formers. However, there was no statistically significant variation in the occurrence rate of strong, intermediate, and non-biofilm-forming *E. faecium* isolates. Sample-wise, the highest percentage of strong biofilm-forming *E. faecium* was found in shrimp (57.1%, 4/7, 95% CI: 25.1–84.2), followed by marine fish (42.3%, 11/26, 95% CI: 25.5–61.1%) and crab (37.5%, 3/8, 95% CI: 13.7–69.4%) samples (Supplementary Table S1).

### 3.3. Prevalence of Virulence Factors in Biofilm-Forming Enterococcus faecium

**Figure 3:**
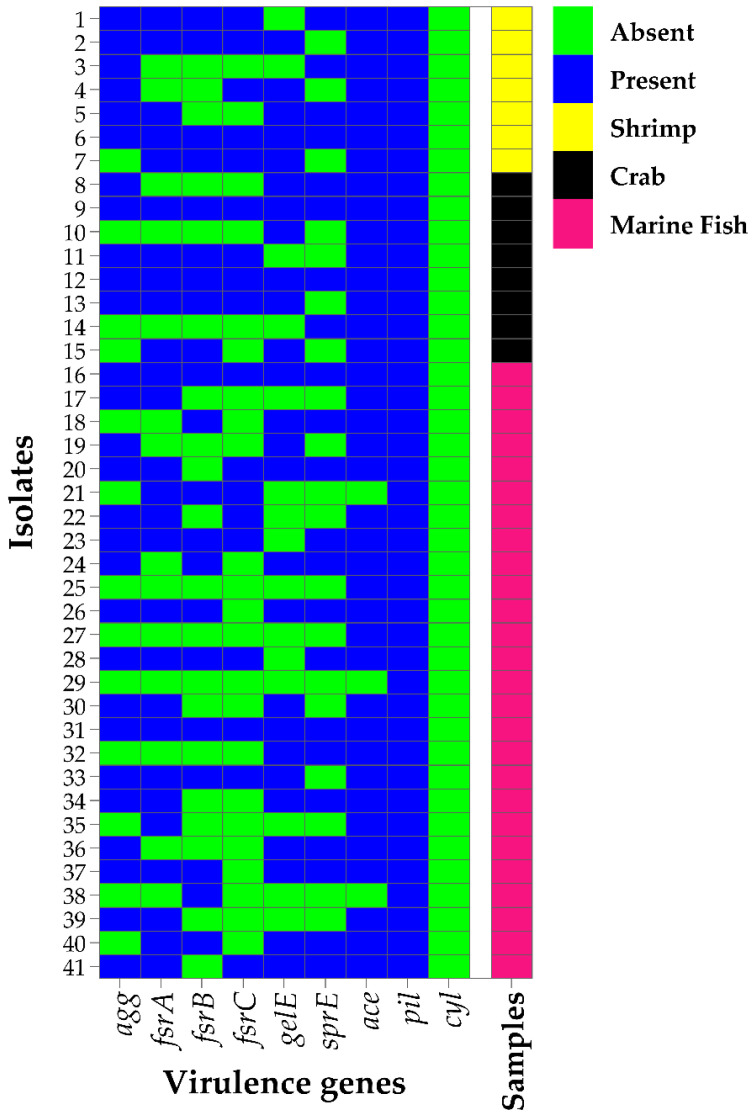
Heatmap showing the virulence profiles of *Enterococcus faecium* isolated from raw seafood in Bangladesh.

In PCR screening, all the *E. faecium* isolates were found to carry at least one of the nine virulence genes tested (Figure 3 and Supplementary Table S1). The highest prevalence of virulence genes was observed for *pil* (100%), followed by *ace* (92.7%), *agg* (68.3%), *fsrA* (65.9%), *gelE* (63.4%), *sprE* (53.7%), *fsrB* (51.2%), and *fsrC* (43.9%). However, none of the *E. faecium* isolates were found to harbor the virulence *cyl* gene (Figure 3 and Table 3).

In bivariate analysis, a strong and positive significant correlation was observed between virulence genes *fsrA* and *fsrC* (Spearman correlation coefficient, ρ = 0.533, *p* < 0.001), *fsrB* and *fsrC* (ρ = 0.470, *p* < 0.002), *fsrA* and *fsrB* (ρ = 0.429, *p* = 0.005), and *ace* and *agg* (ρ = 0.412, *p* = 0.007). A moderate to lower positive significant correlation was also depicted between *agg* and *fsrA* (ρ = 0.394, *p* = 0.011), *agg* and *fsrC* (ρ = 0.392, *p* = 0.011), *ace* and *gelE* (ρ = 0.370, *p* = 0.017), *agg* and *sprE* (ρ = 0.313, *p* = 0.046), and *gelE* and *sprE* (ρ = 0.310, *p* = 0.049) (Supplementary Table S2).

Moreover, the occurrence rate of all the investigated virulence genes (except *pil* and *cyl*) showed a higher trend in the strong biofilm-forming *E. faecium* isolates than in intermediate and non-biofilm formers, i.e., *agg* (strong: 88.9% vs. intermediate: 57.1% vs. non: 44.4%), *fsrA* (88.9% vs. 50% vs. 44.4%), *fsrB* (77.8% vs. 42.9% vs. 11.1%), *fsrC* (72.2%, 28.6% vs. 11.1%), *gelE* (83.3% vs. 57.1% vs. 33.3%), *sprE* (72.2% vs. 50% vs. 22.2%), and *ace* (100% vs. 92.9% vs. 77.8%) (Table 3). In the statistical analysis, the percentage of virulence genes, i.e., *agg*, *fsrA*, *fsrB*, *fsrC*, *gelE*, and *sprE*, was significantly (*p* < 0.05) higher in strong biofilm-forming *E. faecium* isolates than in intermediate and non-biofilm formers (Table 3).

### 3.4. Antibiogram Profiles of Biofilm-Forming Enterococcus faecium

**Figure 4:**
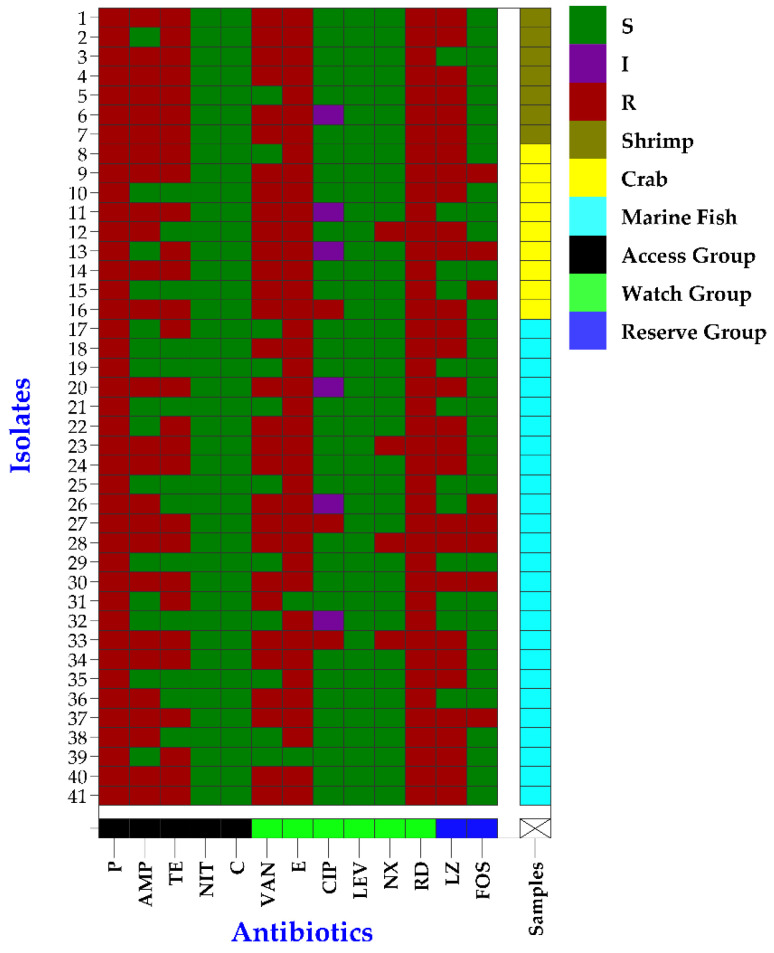
Heatmap exhibiting the antibiogram profiles of *Enterococcus faecium* isolated from raw seafood in Bangladesh; P = penicillin, AMP = ampicillin, TE = tetracycline, NIT = nitrofurantoin, C = chloramphenicol, VA = vancomycin, E = erythromycin, CIP = ciprofloxacin, LEV = levofloxacin, NOR = norfloxacin, RD = rifampin, LZD = linezolid, FOS = fosfomycin.

In AST, *E. faecium* isolates showed resistance to all three WHO-classified antibiotic groups, i.e., access, watch, and reserve (Figure 4). All the *E. faecium* isolates were resistant to at least three antibiotics (Figure 4 and Supplementary Table S1). The highest resistance of *E. faecium* was observed against rifampin and penicillin (100%), followed by erythromycin (95.1%), vancomycin (73.2%), linezolid (70.7%), tetracycline (68.3%), ampicillin (63.4%), fosfomycin (19.5%), norfloxacin (9.8%), and ciprofloxacin (7.3%) (Figure 4 and Table 4). No isolates showed resistance to levofloxacin, nitrofurantoin, and chloramphenicol (Figure 4 and Table 4). Moreover, the beta-lactamase gene *bla*_TEM_ was found in 61% (25/41, 95% CI: 45.7–74.3%) of the *E. faecium* isolates (Table 4).

According to bivariate analysis, a strong and positive significant correlation was found between resistance of *E. faecium* isolates to any of two antibiotics, i.e., tetracycline and linezolid (ρ = 0.483, *p* = 0.001), tetracycline and ampicillin (ρ = 0.462, *p* = 0.002), ampicillin and vancomycin (ρ = 0.454, *p* = 0.003), and vancomycin and tetracycline (ρ = 0.415, *p* = 0.007) (Supplementary Table S3).

Moreover, a statistically significant correlation (*p* < 0.05) between the degrees of biofilm formation and the resistance patterns of *E. faecium* isolates to tetracycline, linezolid, ampicillin, and vancomycin was observed (Table 4). Furthermore, the strong biofilm-forming *E. faecium* isolates showed higher trends of resistance to most of the antibiotics and resistance genes when compared to intermediate and non-biofilm formers, i.e., ciprofloxacin (strong: 11.1% vs. intermediate: 7.1% vs. non: 0%), tetracycline (94.4% vs. 57.1% vs. 33.3%), fosfomycin (33.3% vs. 14.3% vs. 0%), linezolid (88.9% vs. 64.3% vs. 44.4%), norfloxacin (16.7% vs. 7.7% vs. 0%), ampicillin (94.4% vs. 57.1% vs. 11.1%), vancomycin (100% vs. 85.7% vs. 0%), erythromycin (100% vs. 92.9% vs. 88.9%), and resistance *bla*_TEM_ gene (66.7% vs. 64.3% vs. 44.4%) (Table 4).

### 3.5. Phenotypic MDR and MAR Nature in Biofilm-Forming Enterococcus faecium

All the *E. faecium* isolates (95% CI: 91.4–100%) were phenotypically MDR in nature, and a total of 24 resistance patterns were found. The most prevalent MDR pattern, labeled as pattern number 9 (P, AMP, VAN, E, TE, LZ, RD), was found in the highest number of MDR *E. faecium* isolates (9/41, 22%, 95% CI: 12.0–36.7%). Additionally, one isolate demonstrated resistance to eight antimicrobial classes (Pattern 1), encompassing a total of ten antibiotics (Table 5). Furthermore, all 41 *E. faecium* isolates displayed MAR indices greater than 0.2 (MAR index: 0.2–0.8) (Table 5).

## 4. Discussion

Enterococci, primarily due to their ability to withstand harsh environmental conditions during food production processes and their remarkable adaptability to storage conditions, constitute the predominant bacterial group found in food. Research has documented the occurrence of enterococci in seafood sources. Moreover, biofilm formation and the development of AMR in enterococci limit the treatment options for enterococcal infections. To the best of our knowledge, there is currently no information regarding the distribution and diversity of antibiotic resistance and virulence characteristics in biofilm-producing *E. faecium* isolates obtained from seafood. This study provides the initial comprehensive analyses of antibiotic resistance and virulence determinants within *E. faecium* from uncooked seafood.

### 4.1. Enterococcus faecium Isolated from Seafood

In this study, 27.3% (41/150) of the samples were contaminated with *E. faecium*, where the majority of the isolates were detected in sea fish samples. Aligned with our study, Ben Said et al. [5] recorded a similar prevalence, detecting *E. faecium* in 25% of seafood samples. However, the prevalence of *E. faecium* in this study contrasted with previous reports by Valenzuela et al. [16], Boss et al. [28], and Igbinosa and Beshiru [4], in which the detection rate of *E. faecium* in seafood samples was lower than the prevalence recorded in the present study. The variation in the prevalence of *E. faecium* isolates in seafood samples might be due to the geographical location of the studies, environmental conditions, types and numbers of seafood samples collected, and microbial loads in those collected samples or selected areas. The presence of *E. faecium* in seafood samples is not unusual, as several studies have indicated that seafood can become naturally contaminated from the surroundings in which seafood is typically collected [16,29,30]. The findings of the current study also suggest that seafood samples could potentially come into contact with contaminants during the removal of fish entrails and from environmental sources while being processed and handled by individuals. Moreover, the detection of *E. faecium* in seafood is regarded as a signal of fecal or environmental pollution, signifying a potential hazard to human health. Over the last two decades, *E. faecium* has undergone swift evolution into a global nosocomial pathogen, effectively adjusting to the environment within healthcare facilities and giving rise to various human illnesses, including but not limited to neonatal meningitis, endocarditis, bloodstream infections, urinary tract infections, and sepsis among infants [31].

### 4.2. Biofilm Formation of Enterococcus faecium Isolated from Seafood

Biofilm formation significantly impacts enterococci’s pathogenicity by providing an optimal setting for bacterial growth and facilitating genetic element transfer from one strain to another [32]. Biofilm formation can additionally serve as enduring reservoirs of contamination, resulting in hygienic issues within food items. In this study, the CRA test was used to evaluate the biofilm-forming capabilities of isolated *E. faecium* from seafood. Although the CRA test is not a sensitive assay like molecular and whole genome sequencing techniques for evaluating biofilm growth, this assay is widely used by researchers because of its reasonable balance between sensitivity and specificity [33]. In the present study, 78.1% (32/41) of the *E. faecium* isolates were biofilm formers, whereas 43.9% and 34.2% of the isolates were strong and intermediate biofilm formers. Our findings suggest that biofilm-forming *E. faecium* could be detected in seafood samples due to inadequate post-process cleaning. It is important to know that these biofilm-forming bacteria can readily travel extended distances along the production line, leading to equipment failures and food deterioration, and posing health risks if they infiltrate food batches destined for consumers [34]. Moreover, the occurrence of biofilm-forming *E. faecium* in seafood has adverse effects on public health, as biofilm formation promotes the emergence of AMR and virulence in bacterial pathogens [35]. Enterococci exhibit a remarkable ability to create biofilms, a distinct strategy for inducing diseases that allows them to endure in hostile surroundings and remain at the infection site for extended periods [36].

### 4.3. Virulence Profiles of Enterococcus faecium Isolated from Seafood

Bacterial virulence attributes can encompass factors that aid in colonization, such as enhancing bacterial adherence to host cells or invasive elements that promote the invasion of epithelial cells, weakening the immune response. Different cell-wall-incorporated surface proteins contribute to the pathogenic nature of enterococci, including substances that cause aggregation, enterococcal surface proteins, and components that bind to collagen [37]. In this study, each *E. faecium* isolate contained at least one of the examined virulence genes. Although the virulence gene *cyl* was absent in all the isolates, other virulence genes were detected in a high number of isolates. Previously, Valenzuela et al. [16], Hammad et al. [15], Chajęcka-Wierzchowska et al. [38], Boss et al. [28], Ben Said et al. [15], and Igbinosa and Beshiru [4] also detected different virulence traits with a different prevalence rate in *E. faecium* isolates from seafood samples. The substantial presence of virulence genes within *E. faecium* detected in seafood samples signals a significant concern for human well-being. Our findings also imply that seafood might serve as a notable means for transmitting these virulent *E. faecium* strains to both humans and the environment. However, this requires further exploration. Furthermore, a significant rise in the presence of virulence genes was noted in *E. faecium* isolates, demonstrating strong and/or moderate biofilm formation. The present finding indicates that as the extent of biofilm formation in *E. faecium* isolates intensifies, so does their capacity to initiate infections. Nevertheless, further research is necessary to establish the precise connection between the biofilm-forming aptitude of enterococci isolates and the presence of their virulence genes.

### 4.4. Antibiotic Resistance Profiles of Enterococcus faecium Isolated from Seafood

The primary concern with enterococci involves their resistance to antibiotics commonly used as treatments for human patients. In addition, employing antimicrobial agents for disease treatment or prevention may apply selective force to both harmful bacteria and symbiotic microorganisms present in the digestive system of seafood. While antibiotic resistance in enterococci isolates has been documented across different origins in Bangladesh [39,40,41,42,43,44,45,46,47,48], there are a lack of data regarding the prevalence of antibiotic resistance in these bacteria specifically in seafood. In this study, all the 41 *E. faecium* isolates were resistant to ≥3 antimicrobial agents and ≥3 antimicrobial classes (MDR), where a higher resistance was exhibited against penicillin, erythromycin, vancomycin, linezolid, tetracycline, and ampicillin. Moreover, the MAR indices of the isolates ranged between 0.2 and 0.8, and the beta-lactamase gene *bla*_TEM_ was found in 61% of the isolates. The elevated proportion of multidrug resistance and MAR indices in *E. faecium* identified in seafood underscores its significance as a potential reservoir of antibiotic resistance that could potentially spread to humans. Previous studies have demonstrated that *E. faecium* isolated from seafood samples exhibited resistance to various antimicrobial agents [4,5,15,16,28,38].

A standard approach to manage severe enterococcal infections involves combining cell-wall inhibitor antibiotics (penicillin, ampicillin, or vancomycin) with aminoglycosides (streptomycin or gentamicin). However, the concerning aspect is that *E. faecium* detected in seafood exhibited elevated resistance levels: 100% to penicillin, 63.4% to ampicillin, and 73.2% to vancomycin, posing a significant public health concern. Additionally, it is alarming that *E. faecium* isolates showed resistance to the reserve group of antibiotics. In this study, 70.7% and 19.5% of the isolates were resistant to critically important antibiotics, linezolid and fosfomycin, respectively. Previously, Chajęcka-Wierzchowska et al. [38] also found that a higher percentage of enterococci (45.7%) isolated from marine samples were resistant to fosfomycin; however, no resistance to linezolid was observed. The occurrence of *E. faecium* isolates in seafood resistant to linezolid and fosfomycin represents a significant menace to public health. This is because fosfomycin and linezolid are last-resort antibiotics employed to address critical infections arising from MDR enterococci [49,50]. Nonetheless, before arriving at such a crucial inference, additional investigations employing MIC determination and molecular methods should be undertaken. The presence of antibiotic-resistant bacteria in seafood has the potential to pose a public health risk, as they could facilitate the spread of resistance traits along the food chain to other bacteria that hold importance in human clinical contexts. It is conceivable that antibiotic-resistant fecal bacteria originating from domestic sewage or other sources such as animal or fish farming, when released into the sea, could potentially transmit their antibiotic resistance traits to the native fish flora, triggering their propagation and prevalence in the marine ecosystem.

Despite the employment of antibiotics and the host’s immune and inflammatory responses, biofilms persist within the organism owing to biofilm cells’ reduced metabolic activity, quorum sensing, and distinct mechanisms of resilience [51]. Bacteria possessing the capacity to form biofilms exhibited heightened resistance compared to bacteria lacking this potential [52]. In accordance with this statement, strong biofilm-forming *E. faecium* isolates exhibited significantly greater resistance to the majority of tested antibiotics. This indicates a potential connection between the biofilm-forming capacity of *E. faecium* isolates and their antimicrobial resistance. The remarkable antibiotic resistance displayed by *E. faecium* that forms biofilms can be explained by various factors. These include the slower metabolism and growth of biofilm producers, making them naturally less responsive to antibiotics. The composition of the extracellular polymeric substance matrix of the biofilm also restricts antibiotic penetration into its areas. Additionally, the unique physiological attributes of biofilm cells stimulate the activation of multidrug efflux pumps and stress-responsive regulatory systems, nurturing the development of antibiotic resistance [53].

## 5. Conclusions

Antibiotic-resistant biofilm-forming *E. faecium*-carrying virulence genes were detected in certain seafood analyzed in this study. This study, therefore, marks the initial investigation highlighting the significance of certain seafood as a potential source of the antibiotic-resistant biofilm-forming pathogenic *E. faecium* that could affect humans. The findings of this research emphasize the crucial need for rigorous hygiene protocols during the processing, packaging, and storage of seafood in Bangladesh. Moreover, the current study also proposes the adoption of proactive measures to ensure responsible antimicrobial usage across various sectors, including the production of food animals.

## Figures and Tables

**Table 1:** List of primers used for specific detection of *Enterococcus faecium* isolates and their virulence and antibiotic resistance genes.

Target Organism and Determinants	Target Genes	Primer Sequences (5′-3′)	Annealing Tm (°C)	Size (bp)	References
*Enterococcus faecium*	*ddl_E_. _faecium_*	F: GCAAGGCTTCTTAGAGA R: CATCGTGTAAGCTAACTTC	50	550	[19]
Virulence	*Agg*	F: TCTTGGACACGACCCATGAT R: AGAAAGAACATCACCACGAGC	58	413	[11]
	*fsrA*	F: CGTTCCGTCTCTCATAGTTA R: GCAGGATTTGAGGTTGCTAA	53	474	[11]
	*fsrB*	F: TAATCTAGGCTTAGTTCCCAC R: CTAAATGGCTCTGTCGTCTAG	55	428	[11]
	*fsrC*	F: GTGTTTTTGATTTCGCCAGAGA R: TATAACAATCCCCAACCGTG	54	716	[11]
	*gelE*	F: GGTGAAGAAGTTACTCTGAC R: GGTATTGAGTTATGAGGGGC	52	704	[11]
	*sprE*	F: CTGAGGACAGAAGACAAGAAG R: GGTTTTTCTCACCTGGATAG	53	432	[11]
	*Ace*	F: GAATGACCGAGAACGATGGC R: CTTGATGTTGGCCTGCTTCC	58	615	[11]
	*Pil*	F: GAAGAAACCAAAGCACCTAC R: CTACCTAAGAAAAGAAACGCG	53	620	[11]
	*Cyl*	F: TGGCGGTATTTTTACTGGAG R: TGAATCGCTCCATTTCTTC	52	186	[11]
Antibiotic resistance	*bla*_TEM_	F: CATTTCCGTGTCGCCCTTAT R: TCCATAGTTGCCTGACTCCC	56	793	[20]

**Table 2:** Prevalence of *Enterococcus faecium* isolated from different seafoods in Bangladesh.

Type of Samples (N*)	n (%) ^P^ [95% CI] ^Q^	*p*-Value	n* (%) ^A^ [95% CI] ^B^
Shrimp (50)	7 (14 ^a^) [7.0–26.1]	0.034	41 (27.3%) [20.8–34.9]
Crabs (25)	8 (32 ^a,b^) [17.2–51.6]		
Marine fish (75)	26 (34.7 ^b^) [24.9–45.9]		

Here, values with different superscripts differ significantly (*p* < 0.05) within the variable under assessment, N* = category-wise sample number, n = number of sample-wise positive isolates, P = sample-wise prevalence, CI = confidence interval, Q = confidence intervals at 95%, n* = number of overall positive isolates, A = overall prevalence, B = overall confidence interval at 95%.

**Table 3:** Association in the detection of virulence genes and determination of biofilm formation in *E. faecium* (n = 41) isolated from raw seafood in Bangladesh.

Virulence Genes	Virulence in Different Degrees of Biofilm Formation			Total No. of Positive Isolates (%) [95% CI]	*p*-Value
	No. (%) of Strong Biofilm Former (n = 18)	No. (%) of Intermediate Biofilm Former (n = 14)	No. (%) of Non-Biofilm Former (n = 9)		
*agg*	16 (88.9 ^a^)	8 (57.1 ^a,b^)	4 (44.4 ^b^)	28 (68.3) [53.0–80.4]	0.035
*fsrA*	16 (88.9 ^a^)	7 (50 ^b^)	4 (44.4 ^b^)	27 (65.9) [50.6–78.4]	0.022
*fsrB*	14 (77.8 ^a^)	6 (42.9 ^a,b^)	1 (11.1 ^b^)	21 (51.2) [36.5–65.8]	0.004
*fsrC*	13 (72.2 ^a^)	4 (28.6 ^b^)	1 (11.1 ^b^)	18 (43.9) [29.9–59.0]	0.004
*pil*	18 (100 ^a^)	14 (100 ^a^)	9 (100 ^a^)	41 (100) [91.4–100]	NA
*gelE*	15 (83.3 ^a^)	8 (57.1 ^a,b^)	3 (33.3 ^b^)	26 (63.4) [48.1–76.4]	0.033
*sprE*	13 (72.2 ^a^)	7 (50 ^a,b^)	2 (22.2 ^b^)	22 (53.7) [38.8–67.9]	0.046
*ace*	18 (100 ^a^)	13 (92.9 ^a^)	7 (77.8 ^a^)	38 (92.7) [80.6–97.5]	0.112
*cyl*	0 (0 ^a^)	0 (0 ^a^)	0 (0 ^a^)	0 (0) [0.0–8.6]	NA

Here, values with different superscripts differ significantly (*p* < 0.05) within the variable under assessment, CI = confidence interval, NA = not applied.

**Table 4:** Association of antibiotic resistance patterns and biofilm formation in *E. faecium* strains (n = 41) detected in raw seafood in Bangladesh.

Categories	Antibiotics	Antibiotic Resistance in Different Degrees of Biofilm Formation			Total No. of Positive Isolates (%) [95% CI]	*p*-Value
		No. (%) of Strong Biofilm Former (n = 18)	No. (%) of Intermediate Biofilm Former (n = 14)	No. (%) of Non-Biofilm Former (n = 9)		
Phenotypic	CIP	2 (11.1 ^a^)	1 (7.1 ^a^)	0 (0 ^a^)	3 (7.3) [2.5–19.4]	0.579
	TE	17 (94.4 ^a^)	8 (57.1 ^b^)	3 (33.3 ^b^)	28 (68.3) [53.0–80.4]	0.003
	LEV	0 (0 ^a^)	0 (0 ^a^)	0 (0 ^a^)	0 (0) [0.0–8.6]	NA
	FOS	6 (33.3 ^a^)	2 (14.3 ^a^)	0 (0 ^a^)	8 (19.5) [10.2–34.0]	0.100
	RD	18 (100 ^a^)	14 (100 ^a^)	9 (100 ^a^)	41 (100) [91.4–100]	NA
	P	18 (100 ^a^)	14 (100 ^a^)	9 (100 ^a^)	41 (100) [91.4–100]	NA
	LZD	16 (88.9 ^a^)	9 (64.3 ^a,b^)	4 (44.4 ^b^)	29 (70.7) [55.5–82.4]	0.046
	NOR	3 (16.7 ^a^)	1 (7.7 ^a^)	0 (0 ^a^)	4 (9.8) [3.9–22.6]	0.374
	NIT	0 (0 ^a^)	0 (0 ^a^)	0 (0 ^a^)	0 (0) [0.0–8.6]	NA
	AMP	17 (94.4 ^a^)	8 (57.1 ^b^)	1 (11.1 ^b^)	26 (63.4) [48.1–76.4]	<0.001
	C	0 (0 ^a^)	0 (0 ^a^)	0 (0 ^a^)	0 (0) [0.0–8.6]	NA
	VA	18 (100 ^a^)	12 (85.7 ^a^)	0 (0 ^a^)	30 (73.2) [58.1–84.3]	<0.001
	E	18 (100 ^a^)	13 (92.9 ^a^)	8 (88.9 ^a^)	39 (95.1) [83.9–99.1]	0.400
Genotypic	*bla*_TEM_	12 (66.7 ^a^)	9 (64.3 ^a^)	4 (44.4 ^a^)	25 (61.0) [45.7–74.3]	0.511

Here, values with different superscripts differ significantly (*p* < 0.05) within the variable under assessment; CIP = ciprofloxacin, TE = tetracycline, LEV = levofloxacin, FOS = fosfomycin, RD = rifampin, P = penicillin, LZD = linezolid, NOR = norfloxacin, NIT = nitrofurantoin, AMP = ampicillin, C = chloramphenicol, VA = vancomycin, E = erythromycin, CI = confidence interval, NA = not applied.

**Table 5:** Multidrug resistance (MDR) and multiple antibiotic resistance (MAR) profiles of *Enterococcus faecium* isolates detected in raw seafood samples in Bangladesh.

No. of Patterns	Antibiotic Resistance Patterns	No. of Antibiotics (Classes)	No. of Isolates	Overall MDR Isolates (%)	MAR Index
1	P, AMP, VAN, E, TE, CIP, NX, LZ, FOS, RD	10 (8)	1	41 (100)	0.8
2	P, AMP, VAN, E, TE, NX, LZ, FOS, RD	9 (8)	1		0.7
3	P, AMP, VAN, E, TE, CIP, LZ, FOS, RD	9 (8)	1		
4	P, AMP, VAN, E, TE, CIP, NX, LZ, RD	9 (7)	1		
5	P, AMP, VAN, E, TE, NX, LZ, RD	8 (7)	1		0.6
6	P, AMP, VAN, E, TE, CIP, LZ, RD	8 (7)	1		
7	P, AMP, VAN, E, TE, LZ, FOS, RD	8 (7)	2		
8	P, VAN, E, TE, LZ, FOS, RD	7 (7)	1		0.5
9	P, AMP, VAN, E, TE, LZ, RD	7 (6)	9		
10	P, AMP, VAN, E, NX, LZ, RD	7 (6)	1		
11	P, VAN, E, TE, LZ, RD	6 (6)	3		0.5
12	P, VAN, E, TE, FOS, RD	6 (6)	1		
13	P, AMP, VAN, E, TE, RD	6 (5)	3		
14	P, AMP, E, TE, LZ, RD	6 (5)	2		
15	P, AMP, VAN, E, LZ, RD	6 (5)	1		
16	P, VAN, E, LZ, RD	5 (5)	1		0.4
17	P, E, TE, LZ, RD	5 (5)	1		
18	P, AMP, VAN, TE, RD	5 (4)	1		
19	P, AMP, VAN, E, RD	5 (4)	1		
20	P, AMP, E, LZ, RD	5 (4)	1		
21	P, E, TE, RD	4 (4)	1		0.3
22	P, E, LZ, RD	4 (4)	1		
23	P, TE, LZ, RD	4 (4)	1		
24	P, E, RD	3 (3)	4		0.2

Here, MDR = multidrug-resistance, MAR = multiple antibiotic resistance, CIP = ciprofloxacin, TE = tetracycline, LEV = levofloxacin, FOS = fosfomycin, RD = rifampin, P = penicillin, LZD = linezolid, NOR = norfloxacin, NIT = nitrofurantoin, AMP = ampicillin, C = chloramphenicol, VA = vancomycin, E = erythromycin.

## Data Availability

All the data are available in the manuscript and Supplementary Material File.

## Supplementary Materials

The following supporting information can be downloaded at: https://www.mdpi.com/article/10.3390/pathogens12091101/s1, Table S1: Antibiotic resistance, biofilm, and virulence profiles of *Enterococcus faecium* isolated from seafood in Bangladesh; Table S2: Pearson correlation coefficient to assess the pairs of any of two virulence genes detected in *E. faecium* isolated from seafood in Bangladesh; Table S3: Pearson correlation coefficients between any of two antibiotics to which *E. faecium* isolates showed resistance.

## References

[1] Islam M.M., Shamsuzzaman M.M., Mozumder M.M.H., Xiangmin X., Ming Y., Jewel M.A.S. (2017). Exploitation and conservation of coastal and marine fisheries in Bangladesh: Do the fishery laws matter?. Mar. Policy.

[2] Department of Fisheries (DoF) Yearbook of Fisheries Statistics of Bangladesh, 2021–2022, Fisheries Resources Survey System (FRSS), 2023, Volume 39, Ministry of Fisheries, Bangladesh. http://fisheries.portal.gov.bd/site/download/42836060-aa5e-491d-8309-cf750886813b.

[3] Novoslavskij A., Terentjeva M., Eizenberga I., Valciņa O., Bartkevičs V., Bērziņš A. (2016). Major food borne pathogens in fish and fish products: A review. Ann. Microbiol..

[4] Igbinosa E.O., Beshiru A. (2019). Antimicrobial resistance, virulence determinants, and biofilm formation of Enterococcus species from ready-to-eat seafood. Front. Microbiol..

[5] Ben Said L., Hamdaoui M., Klibi A., Ben Slama K., Torres C., Klibi N. (2017). Diversity of species and antibiotic resistance in enterococci isolated from seafood in Tunisia. Ann. Microbiol..

[6] Ramos S., Silva V., Dapkevicius M.D.L.E., Igrejas G., Poeta P. (2020). Enterococci, from harmless bacteria to a pathogen. Microorganisms.

[7] Torres C., Alonso C.A., Ruiz-Ripa L., León-Sampedro R., Del Campo R., Coque T.M. (2018). Antimicrobial resistance in *Enterococcus* spp. of animal origin. Microbiol. Spectr..

[8] ECDC (2011). European Centre for Disease Prevention and Control Publishes Annual Epidemiological Report 2011. Euro. Surveill..

[9] Costa O.Y., Raaijmakers J.M., Kuramae E.E. (2018). Microbial extracellular polymeric substances: Ecological function and impact on soil aggregation. Front. Microbiol..

[10] Souza E.L., Meira Q.G., Barbosa I.D., Athayde A.J., Conceição M.L., Siqueira Júnior J.P. (2014). Biofilm formation by *Staphylococcus aureus* from food contact surfaces in a meat-based broth and sensitivity to sanitizers. Braz. J. Microbiol..

[11] Hashem Y.A., Amin H.M., Essam T.M., Yassin A.S., Aziz R.K. (2017). Biofilm formation in enterococci: Genotype-phenotype correlations and inhibition by vancomycin. Sci. Rep..

[12] Hancock L.E., Gilmore M.S., Fischetti V.A., Novick R.P., Ferretti J.J., Portnoy D.A., Rood J.I. (2006). Pathogenicity of enterococci. Gram-Positive Pathogens.

[13] Prestinaci F., Pezzotti P., Pantosti A. (2015). Antimicrobial resistance: A global multifaceted phenomenon. Pathog. Glob. Health.

[14] Michael C.A., Dominey-Howes D., Labbate M. (2014). The Antimicrobial Resistance Crisis: Causes, Consequences, and Management. Front. Public Health.

[15] Hammad A.M., Shimamoto T., Shimamoto T. (2014). Genetic characterization of antibiotic resistance and virulence factors in *Enterococcus* spp. From Japanese retail ready-to-eat raw fish. Food Microbiol..

[16] Valenzuela A.S., Benomar N., Abriouel H., Cañamero M.M., Gálvez A. (2010). Isolation and identification of *Enterococcus faecium* from seafoods: Antimicrobial resistance and production of bacteriocin-like substances. Food Microbiol..

[17] Samia S., Galib H.T., Tanvir A.S., Basudeb C.S., Walliullah M., Tasnia A., Sakil M.M., Afsana F.N., Sadia K.P., Kamal K.D. (2014). Microbiological quality analysis of shrimps collected from local market around Dhaka city. Int. Food Res. J..

[18] Facklam R.R., da Carvalho M.G.S., Teixeira L.M. (2002). History, Taxonomy, Biochemical Characteristics, and Antibiotic Susceptibility Testing of Enterococci. The Enterococci.

[19] Dutka-Malen S., Evers S., Courvalin P. (1995). Detection of glycopeptide resistance genotypes identification to the species level of clinically relevant enterococci by PCR. J. Clin. Microbiol..

[20] Randall L.P., Cooles S.W., Osborn M.K., Piddock L.J.V., Woodward M.J. (2004). Antibiotic resistance genes integrons multiple antibiotic resistance in thirty-five serotypes of *Salmonella enterica* isolated from humans animals in the UK. J. Antimicrob. Chemother..

[21] Queipo-Ortuno M.I., De Dios Colmenero J., Macias M., Bravo J.M., Morata P. (2008). Preparation of bacterial DNA template by boiling and effect of immunoglobulin G as an inhibitor in real-time PCR for serum samples from patients with brucellosis. Clin. Vaccine Immunol..

[22] Zheng J.X., Bai B., Lin Z.W., Pu Z.Y., Yao W.M., Chen Z., Li D.Y., Deng X.B., Deng Q.W., Yu Z.J. (2018). Characterization of biofilm formation by *Enterococcus faecalis* isolates derived from urinary tract infections in China. J. Med. Microbiol..

[23] (2022). Performance Standards for Antimicrobial Susceptibility Testing.

[24] Bauer A.T., Kirby W.M.M., Sherris J.C., Turck M. (1966). Antibiotic susceptibility testing by a standardized single disc method. Am. J. Clin. Pathol..

[25] Magiorakos A.P., Srinivasan A., Carey R.B., Carmeli Y., Falagas M.E., Giske C.G., Harbarth S., Hindler J.F., Kahlmeter G., Olsson-Liljequist B. (2012). Multidrug-resistant, extensively drug-resistant and pandrug-resistant bacteria: An international expert proposal for interim standard definitions for acquired resistance. Clin. Microbiol. Infect..

[26] Krumperman P.H. (1983). Multiple antibiotic resistance indexing of *Escherichia coli* to identify high-risk sources of fecal contamination of foods. Appl. Environ. Microbiol..

[27] Brown L.D., Cai T.T., DasGupta A. (2001). Interval estimation for a binomial proportion. Stat. Sci..

[28] Boss R., Overesch G., Baumgartner A. (2016). Antimicrobial Resistance of *Escherichia coli*, Enterococci, *Pseudomonas aeruginosa*, and *Staphylococcus aureus* from Raw Fish and Seafood Imported into Switzerland. J. Food Protect..

[29] Çardak M., Özmen Toğay S., Ay M., Karaalioğlu O., Erol Ö., Bağcı U. (2022). Antibiotic resistance and virulence genes in Enterococcus species isolated from raw and processed seafood. J. Food Sci. Technol..

[30] Chotinantakul K., Chansiw N., Okada S. (2018). Antimicrobial resistance of Enterococcus spp. isolated from Thai fermented pork in Chiang Rai Province, Thailand. J. Glob. Antimicrob. Resist..

[31] Zhou X., Willems R.J., Friedrich A.W., Rossen J.W., Bathoorn E. (2020). *Enterococcus faecium*: From microbiological insights to practical recommendations for infection control and diagnostics. Antimicrob. Resist. Infect. Control.

[32] Sieńko A., Wieczorek P., Majewski P., Ojdana D., Wieczorek A., Olszańska D., Tryniszewska E. (2015). Comparison of antibiotic resistance and virulence between biofilm-producing and non-producing clinical isolates of *Enterococcus faecium*. Acta Biochim. Pol..

[33] Hentzer M., Riedel K., Rasmussen T.B., Heydorn A., Andersen J.B., Parsek M.R., Rice S.A., Eberl L., Molin S., Høiby N. (2002). Inhibition of quorum sensing in *Pseudomonas aeruginosa* biofilm bacteria by a halogenated furanone compound. Microbiology.

[34] Gajewska J., Chajęcka-Wierzchowska W., Byczkowska-Rostkowska Z., Saki M. (2023). Biofilm Formation Capacity and Presence of Virulence Determinants among *Enterococcus* Species from Milk and Raw Milk Cheeses. Life.

[35] Liu X., Yao H., Zhao X., Ge C. (2023). Biofilm Formation and Control of Foodborne Pathogenic Bacteria. Molecules.

[36] Tibúrcio A.A.C.M., Paiva A.D., Pedrosa A.L., Rodrigues W.F., da Silva R.B., Oliveira A.G. (2022). Effect of sub-inhibitory concentrations of antibiotics on biofilm formation and expression of virulence genes in penicillin-resistant, ampicillin-susceptible *Enterococcus faecalis*. Heliyon.

[37] Hendrickx A.P., Willems R.J., Bonten M.J., van Schaik W. (2009). LPxTG surface proteins of enterococci. Trends Microbiol..

[38] Roy K., Islam M.S., Paul A., Ievy S., Talukder M., Sobur M.A., Ballah F.M., Khan M.S.R., Rahman M.T. (2022). Molecular detection and antibiotyping of multi-drug resistant *Enterococcus faecium* from healthy broiler chickens in Bangladesh. Vet. Med. Sci..

[39] Chajęcka-Wierzchowska W., Zadernowska A., Łaniewska-Trokenheim Ł. (2016). Virulence factors, antimicrobial resistance and biofilm formation in *Enterococcus* spp. isolated from retail shrimps. LWT-Food Sci. Technol..

[40] Banik A., Mohammad N., Akter T., Fatema K., Abony M. (2018). Prevalence, identification and antibiotic susceptibility of *Enterococcus* Species isolated from chicken and pigeon meat in Gazipur area of Bangladesh. Open J. Med. Microbiol..

[41] Roy S., Aung M.S., Paul S.K., Ahmed S., Haque N., Khan E.R., Barman T.K., Islam A., Abedin S., Sultana C. (2020). Drug resistance determinants in clinical isolates of *Enterococcus faecalis* in Bangladesh: Identification of oxazolidinone resistance gene optrA in ST59 and ST902 lineages. Microorganisms.

[42] Akter T., Foysal M.J., Alam M., Ehsan R., Paul S.I., Momtaz F., Siddik M.A., Tay A.C.Y., Fotedar R., Gupta S.K. (2021). Involvement of *Enterococcus* species in streptococcosis of Nile tilapia in Bangladesh. Aquaculture.

[43] Islam M.S., Paul A., Talukder M., Roy K., Sobur M.A., Ievy S., Nayeem M.M.H., Rahman S., Nazir K.N.H., Hossain M.T. (2021). Migratory birds travelling to Bangladesh are potential carriers of multi-drug resistant *Enterococcus* spp., *Salmonella* spp., and *Vibrio* spp. Saudi J. Biol. Sci..

[44] Sagor M.S., Hossain M.S., Islam T., Mahmud M.A., Miah M.S., Karim M.R., Giasuddin M., Samad M.A. (2022). Phenotypic and genotypic antibiotic resistance and virulence profiling of *Enterococcus faecalis* isolated from poultry at two major districts in Bangladesh. Pak. Vet. J..

[45] Bag M.A.S., Arif M., Riaz S., Khan M.S.R., Islam M.S., Punom S.A., Ali M.W., Begum F., Islam M.S., Rahman M.T. (2022). Antimicrobial resistance, virulence profiles, and public health significance of *Enterococcus faecalis* isolated from clinical mastitis of cattle in Bangladesh. BioMed Res. Int..

[46] Samad M.A., Sagor M.S., Hossain M.S., Karim M.R., Mahmud M.A., Sarker M.S., Shownaw F.A., Mia Z., Card R.M., Agunos A. (2022). High prevalence of vancomycin non-susceptible and multi-drug resistant enterococci in farmed animals and fresh retail meats in Bangladesh. Vet. Res. Commun..

[47] Akter T., Haque M.N., Ehsan R., Paul S.I., Foysal M.J., Tay A.C.Y., Islam M.T., Rahman M.M. (2023). Virulence and antibiotic-resistance genes in *Enterococcus faecalis* associated with streptococcosis disease in fish. Sci. Rep..

[48] Ferdous F.B., Islam M.S., Ullah M.A., Rana M.L., Punom S.A., Neloy F.H., Chowdhury M.N.U., Hassan J., Siddique M.P., Saha S. (2023). Antimicrobial Resistance Profiles, Virulence Determinants, and Biofilm Formation in Enterococci Isolated from Rhesus Macaques (*Macaca mulatta*): A Potential Threat for Wildlife in Bangladesh?. Animals.

[49] Fioriti S., Coccitto S.N., Cedraro N., Simoni S., Morroni G., Brenciani A., Mangiaterra G., Vignaroli C., Vezzulli L., Biavasco F. (2021). Linezolid resistance genes in enterococci isolated from sediment and zooplankton in two Italian coastal areas. Appl. Environ. Microbiol..

[50] Abbott I.J., van Gorp E., van der Meijden A., Wijma R.A., Meletiadis J., Roberts J.A., Mouton J.W., Peleg A.Y. (2020). Oral fosfomycin treatment for enterococcal urinary tract infections in a dynamic in vitro model. Antimicrob. Agents Chemother..

[51] Høiby N., Bjarnsholt T., Givskov M., Molin S., Ciofu O. (2010). Antibiotic resistance of bacterial biofilms. Int. J. Antimicrob. Agents.

[52] Uruén C., Chopo-Escuin G., Tommassen J., Mainar-Jaime R.C., Arenas J. (2020). Biofilms as promoters of bacterial antibiotic resistance and tolerance. Antibiotics.

[53] Davies D. (2003). Understanding biofilm resistance to antibacterial agents. Nat. Rev. Drug Discov..

