# Supplementary material for: Resistance Profiles and Virulence Determinants in Biofilm-Forming Enterococcus faecium Isolated from Raw Seafood in Bangladesh
Source: Pathogens. 2023 Aug 28;12(9):1101. doi: 10.3390/pathogens12091101 (PMC10535238; doi:10.3390/pathogens12091101)
Supplement: Supplementary file 1 [file pathogens-12-01101-s001.zip › pathogens-2577309-supplementary.pdf]

Table S1. Antibiotic resistance, biofilm, and virulence profiles of *Enterococcus faecium* isolated from seafood in Bangladesh.

SL No.	Sample Types	Sample ID	Biofilm Properties	Antibiogram Profiles		Virulence Gene Patterns
				Phenotypic	Genotypic	
**01**	Shrimp	WSM1	Strong	P, AMP, VAN, E, TE, LZ, RD	bla_{TEM}	*agg, fsrA, fsrB, fsrC, sprE, ace, pil*
02		WSM7	Intermediate	P, VAN, E, TE, LZ, RD		*agg, fsrA, fsrB, fsrC, gelE, ace, pil*
03		WSC3	Intermediate	P, AMP, VAN, E, TE, RD	bla_{TEM}	*agg, sprE, ace, pil*
04		WSC10	Strong	P, AMP, VAN, E, TE, LZ, RD	bla_{TEM}	*agg, fsrC, gelE, ace, pil*
05		WSK7	Intermediate	P, AMP, E, TE, LZ, RD	bla_{TEM}	*agg, fsrA, gelE, sprE, ace, pil*
06		TSM5	Strong	P, AMP, VAN, E, TE, LZ, RD	bla_{TEM}	*agg, fsrA, fsrB, fsrC, gelE, sprE, ace, pil*
07		TSC8	Strong	P, AMP, VAN, E, TE, LZ, RD		*fsrA, fsrB, fsrC, gelE, ace, pil*
08	Crab	CM5	Non –former	P, AMP, E, TE, LZ, RD	bla_{TEM}	*agg, gelE, sprE, ace, pil*
09		CM8	Strong	P, AMP, VAN, E, TE, LZ, FOS, RD	bla_{TEM}	*agg, fsrA, fsrB, fsrC, gelE, sprE, ace, pil*
10		CM09	Intermediate	P, VAN, E, LZ, RD	bla_{TEM}	*gelE, ace, pil*
11		CC02	Intermediate	P, AMP, VAN, E, TE, RD	bla_{TEM}	*agg, fsrA, fsrB, fsrC, ace, pil*
12		CC07	Strong	P, AMP, VAN, E, NX, LZ, RD	bla_{TEM}	*agg, fsrA, fsrB, fsrC, gelE, sprE, ace, pil*
13		CC10	Strong	P, VAN, E, TE, LZ, FOS, RD	bla_{TEM}	*agg, fsrA, fsrB, fsrC, gelE, ace, pil*
14		CK01	Intermediate	P, AMP, VAN, E, TE, RD		*sprE, ace, pil*
15		CK05	Intermediate	P, VAN, E, TE, FOS, RD		*fsrA, fsrB, gelE, ace, pil*
16	Marine Fish	RM02	Strong	P, AMP, VAN, E, TE, CIP, LZ, RD	bla_{TEM}	*agg, fsrA, fsrB, fsrC, gelE, sprE, ace, pil*
17		RM07	Non –former	P, E, TE, LZ, RD		*agg, fsrA, ace, pil*
18		RM10	Intermediate	P, VAN, E, TE, LZ, RD		*fsrB, gelE, sprE, ace, pil*
19		RC01	Non –former	P, E, TE, RD		*agg, gelE, ace, pil*
20		RC04	Strong	P, AMP, VAN, E, TE, LZ, RD	bla_{TEM}	*agg, fsrA, fsrC, gelE, sprE, ace, pil*
21		RK01	Non –former	P, E, RD		*Pil, fsrA, fsrB, fsrC*
22		RK02	Intermediate	P, VAN, E, TE, LZ, RD	bla_{TEM}	*agg, fsrA, fsrC, ace, pil*
23		TM01	Strong	P, AMP, VAN, E, TE, NX, LZ, RD	bla_{TEM}	*agg, fsrA, fsrB, fsrC, sprE, ace, pil*
24		TM02	Strong	P, AMP, VAN, E, TE, LZ, RD	bla_{TEM}	*agg, fsrB, gelE, sprE, ace, pil*
25		TM05	Non –former	P, E, RD		*ace, pil*
26		TM08	Intermediate	P, AMP, VAN, E, LZ, RD	bla_{TEM}	*agg, fsrA, fsrB, gelE, sprE, ace, pil*
27		TM10	Intermediate	P, AMP, VAN, E, TE, CIP, LZ, FOS, RD	bla_{TEM}	*ace, pil*
28		TC04	Strong	P, AMP, VAN, E, TE, CIP, NX, LZ, FOS, RD	bla_{TEM}	*agg, fsrA, fsrB, fsrC, sprE, ace, pil*
29		TC07	Non –former	P, E, RD		*pil*

30		TK02	Strong	P, AMP, VAN, E, TE, LZ, FOS, RD	bla_{TEM}	*agg, fsrA, gelE, ace, pil*
31		TK03	Intermediate	P, AMP, VAN, TE, RD		*agg, fsrA, fsrB, fsrC, gelE, sprE, ace, pil*
32		TK05	Non –former	P, E, RD		*gelE, sprE, ace, pil*
33		LM03	Strong	P, AMP, VAN, E, TE, CIP, NX, LZ, RD	bla_{TEM}	*agg, fsrA, fsrB, fsrC, gelE, ace, pil*
34		LM09	Strong	P, AMP, VAN, E, TE, LZ, RD		*agg, fsrA, gelE, sprE, ace, pil*
35		LM10	Non –former	P, E, LZ, RD	bla_{TEM}	*fsrA, ace, pil*
36		LC01	Intermediate	P, AMP, VAN, E, RD	bla_{TEM}	*agg, gelE, sprE, ace, pil*
37		LC03	Strong	P, AMP, VAN, E, TE, NX, LZ, FOS, RD		*agg, fsrA, fsrB, gelE, sprE, ace, pil*
38		LC08	Intermediate	P, AMP, E, LZ, RD	bla_{TEM}	*fsrB, pil*
39		LK01	Non –former	P, TE, LZ, RD		*agg, fsrA, ace, pil*
40		LK02	Strong	P, AMP, VAN, E, TE, LZ, RD	bla_{TEM}	*fsrA, fsrB, gelE, sprE, ace, pil*
41		LK04	Strong	P, AMP, VAN, E, TE, LZ, RD		*agg, fsrA, fsrC, gelE, sprE, ace, pil*

Here, CIP = Ciprofloxacin, TE = Tetracycline, LEV = Levofloxacin, FOS = Fosfomycin, RD = Rifampin, P = Penicillin, LZD = Linezolid, NOR = Norfloxacin, NIT = Nitrofurantoin, AMP = Ampicillin, C = Chloramphenicol, VA = Vancomycin, E = Erythromycin.

Table S2: Pearson correlation coefficient to assess the pairs of any of two virulence genes detected in *E. faecium* isolated from seafood in Bangladesh.

		agg	*fsrA*	*fsrB*	*fsrC*	*gelE*	*sprE*	*ace*	*pil*	*cyl*
agg	Pearson Correlation	1								
	Sig. (2-tailed)									
fsrA	Pearson Correlation	**.394***	1							
	Sig. (2-tailed)	0.011								
fsrB	Pearson Correlation	0.069	**.429****	1						
	Sig. (2-tailed)	0.668	0.005							
fsrC	Pearson Correlation	**.392***	**.533****	**.470****	1					
	Sig. (2-tailed)	0.011	0	0.002						
gelE	Pearson Correlation	0.244	0.094	0.17	0.06	1				
	Sig. (2-tailed)	0.124	0.56	0.287	0.711					
sprE	Pearson Correlation	**.313***	0.053	0.169	0.034	**.310***	1			
	Sig. (2-tailed)	0.046	0.743	0.29	0.835	0.049				
ace	Pearson Correlation	**.412****	0.193	-0.087	0.06	**.370***	0.302	1		
	Sig. (2-tailed)	0.007	0.227	0.589	0.71	0.017	0.055			
pil	Pearson Correlation	.c	.c	.c	.c	.c	.c	.c	.c	
	Sig. (2-tailed)	.	.	.	.	.	.	.		.
cyl	Pearson Correlation	.c	.c	.c	.c	.c	.c	.c	.c	.c
	Sig. (2-tailed)	.	.	.	.	.	.	.	.	

*Correlation is significant at the 0.05 level (2-tailed); **Correlation is significant at the 0.01 level (2-tailed); [c]Cannot be computed because at least one of the variables is constant.

Table S3. Pearson correlation coefficients between any of two antibiotics to which *E. faecium* isolates showed resistance.

		P	AMP	VAN	E	TE	CIP	LEV	NX	NIT	C	LZ	FOS	RD
**P**	Pearson Correlation	.a												
	Sig. (2-tailed)		.											
**AMP**	Pearson Correlation	.a	1											
	Sig. (2-tailed)	.												
**VAN**	Pearson Correlation	.a	**.454****	1										
	Sig. (2-tailed)	.	0.003											
**E**	Pearson Correlation	.a	0.298	0.118	1									
	Sig. (2-tailed)	.	0.058	0.461										
**TE**	Pearson Correlation	.a	**.462****	**.415****	-0.154	1								
	Sig. (2-tailed)	.	0.002	0.007	0.335									
**CIP**	Pearson Correlation	.a	0.213	0.17	0.064	0.191	1							
	Sig. (2-tailed)	.	0.18	0.288	0.693	0.23								
**LEV**	Pearson Correlation	.a	.a	.a	.a	.a	.a	.a						
	Sig. (2-tailed)	.	.	.	.	.	.		.					
**NX**	Pearson Correlation	.a	0.25	0.199	0.074	0.047	0.223	.a	1					
	Sig. (2-tailed)	.	0.115	0.212	0.644	0.769	0.161	.		.				
**NIT**	Pearson Correlation	.a	.a	.a	.a	.a	.a	.a	.a	.a				
	Sig. (2-tailed)	.	.	.	.	.	.	.	.					
**C**	Pearson Correlation	.a	.a	.a	.a	.a	.a	.a	.a	.a	.a			
	Sig. (2-tailed)	.	.	.	.	.	.	.	.	.		.		
**LZ**	Pearson Correlation	.a	0.29	0.215	0.103	**.483****	0.181	.a	0.212	.a	.a	1		
	Sig. (2-tailed)	.	0.065	0.176	0.521	0.001	0.258	.	0.184	.	.			
**FOS**	Pearson Correlation	.a	0.118	0.298	0.111	0.071	0.098	.a	0.046	.a	.a	0.046	1	
	Sig. (2-tailed)	.	0.461	0.058	0.488	0.659	0.542	.	0.777	.	.	0.774		.
**RD**	Pearson Correlation	.a	.a	.a	.a	.a	.a	.a	.a	.a	.a	.a	.a	.a
	Sig. (2-tailed)	.	.	.	.	.	.	.	.	.	.	.	.	

** Correlation is significant at the 0.01 level (2-tailed), [a]Cannot be computed because at least one of the variables is constant, CIP = Ciprofloxacin, TE = Tetracycline, LEV = Levofloxacin, FOS = Fosfomycin, RD = Rifampin, P = Penicillin, LZD = Linezolid, NOR = Norfloxacin, NIT = Nitrofurantoin, AMP = Ampicillin, C = Chloramphenicol, VA = Vancomycin, E = Erythromycin.
